# Integrating Osteopathic Principles for Enhanced Dermatological Outcomes: A Literature Review

**DOI:** 10.7759/cureus.52665

**Published:** 2024-01-21

**Authors:** Nina Ventura, Varun Soti

**Affiliations:** 1 Medicine, Lake Erie College of Osteopathic Medicine, Elmira, USA; 2 Pharmacology and Therapeutics, Lake Erie College of Osteopathic Medicine, Elmira, USA

**Keywords:** micro-needling, bullous pemphigoid, managing atopic dermatitis, tenets of osteopathic medicine, dermatology

## Abstract

This paper explores the impact of osteopathic medicine's principles and philosophy on dermatology conditions, focusing specifically on atopic dermatitis (AD), bullous pemphigoid (BP), and acne scars. The aim is to investigate how integrating osteopathic principles into dermatology can improve patient outcomes by addressing visible and internal health factors. The review was conducted through a literature search utilizing PubMed and Journal Storage. By focusing on the interconnectedness of mind, body, and spirit, osteopathic medicine could contribute to the effective treatment of AD. Stress management techniques have been found to significantly aid in the treatment of AD, as stress levels and social stress have a significant impact on the exacerbation of AD symptoms. Micro-needling is a promising treatment for atrophic acne scars, reducing scar severity scores by up to 68.3%. Combining micro-needling with trichloroacetic acid or non-ablative fractional laser technology further enhances treatment efficacy. The development of BP has been linked to alterations in the epidermis. Granzyme B has been identified as a contributing factor in dermal-epidermal junction separation and autoantibody formation, leading to BP. However, the specific link between osteopathic manipulation and Granzyme B levels in BP is not yet firmly established.

Although osteopathic manipulation may impact the immune system and inflammation, further investigation is required to determine its precise effects on granzyme B and BP. Nonetheless, integrating osteopathic principles and philosophy into dermatology can improve patient outcomes by addressing visible and internal health factors. The benefits of such integration include improved patient-provider relationships, innovative treatments, better stress management, and individualized care plans. Practitioners should be educated on the significance of complete skin examinations for all patients, and future research should focus on exploring the benefits of osteopathic manipulation for dermatologic conditions. Further investigations into new dermatological treatment methods rooted in osteopathic principles are encouraged. The foundation of dermatology and osteopathic medicine share the importance of physical touch for diagnosis and treatment. An osteopathic approach to dermatology considers the link between cutaneous diseases and systemic health. This approach aligns with the four fundamental osteopathic beliefs: the body functions as a whole unit; a person is an integration of body, mind, and spirit that cannot be separated. The body can regulate itself, heal itself, and maintain its health. The body’s structure and function are interdependent and work together. Rational treatment is based upon an understanding of the basic principles of body unity, self-regulation, and the interrelationship of structure and function.

## Introduction and background

Dermatology is a multidimensional specialty that centers around treating the whole person. The foundation of dermatology is inherently osteopathic, where physicians establish a deep connection between the individual and their well-being. They recognize the importance of health across all dimensions, not just those visible on the skin. They take a comprehensive approach to treatment that encompasses physical, mental, emotional, and spiritual health [1].

This paper aims to explore integrating osteopathic principles into dermatology, promoting a holistic approach to patient care. By understanding that disease is multifactorial, physicians can apply the four tenets of osteopathic medicine to treat the person as a whole. These tenets are paired with a dermatologic parallel to illustrate the practical applications of osteopathic principles within dermatology [1].

Andrew Taylor Still developed the philosophy of osteopathic medicine, which recognizes health’s physical, mental, emotional, and spiritual components. He believed that somatic dysfunctions, or musculoskeletal impediments, are readily accessible to the hands and responsive to osteopathic manipulative treatments. By taking a deeper look beneath the skin and assessing the person's overall health, physicians can provide comprehensive treatment that addresses all aspects of health. There are four core tenets recognized by the American Osteopathic Association (AOA) that underline the philosophy of osteopathic medicine [1]: 1. The body is a unit; the person is a unit of body, mind, and spirit. 2. The body is capable of self-regulation, self-healing, and health maintenance. 3. Structure and function are reciprocally interrelated. 4. Rational treatment is based on understanding the basic principles of body unity, self-regulation, and the interrelationship of structure and function.

Dermatologists play a crucial role in identifying cutaneous signs of internal disease. By understanding how systemic diseases impact the entire body, they can discern various skin symptoms that can help with early diagnosis and treatment. Dermatologists with additional training in osteopathic medicine can use somatic findings to establish a link between the body’s structure and function. This can be further enhanced by using osteopathic manipulative treatment to promote homeostatic mechanisms in the body. Effective treatment can not only help alleviate skin symptoms but may also eliminate the need for systemic therapy, which can be more expensive and time-consuming. Overall, dermatologists can provide valuable insights into the underlying causes of cutaneous manifestations and offer treatment options that can lead to better patient outcomes [2-3]. 

## Review

Methods

**Figure 1:**
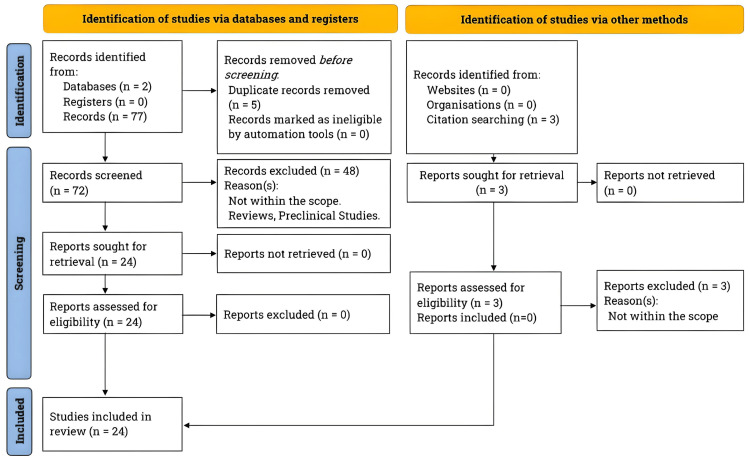
The PRISMA flowchart illustrating the literature search and study selection process. Per the PRISMA guidelines, we searched for clinical studies on applying osteopathic principles to dermatological conditions. Our focus was mainly on stress, itch, atopic dermatitis, psychological impact, percutaneous collagen induction, skin injury, healing, skin structure, and function. The search was conducted on PubMed and Journal Storage from October 2022 to October 2023, specifically focusing on studies published in the last five decades. PRISMA, Preferred Reporting Items for Systematic Reviews and Meta-Analyses.

A comprehensive literature review was conducted following the Preferred Reporting Items for Systematic Reviews and Meta-Analyses (PRISMA) guidelines [4]. A search was conducted between October 2022 and October 2023 using PubMed and Journal Storage. The inclusion criteria were original research conducted on human subjects and published in English. Twenty-four studies were included, with 10 focusing on the first tenet of osteopathic medicine, eight on the second, and six on the third. The first tenet was examined using search terms "Stress," "Itch," "Atopic Dermatitis," and associated "Psychological Impact." For the second tenet, "Percutaneous Collagen Induction" was searched, focusing on how the induction of skin injury can induce healing. The third tenet concentrated on "Skin Structure," "Function," and "Integrity" concerning "Bullous Pemphigoid." Finally, the fourth tenet tied together all the discussed concepts and the importance of looking for the "Cutaneous Signs of Systemic Disease." Please refer to Figure 1, which shows the PRISMA flowchart for literature search and study selection.

Results

Tenet One: The Body Is a Unit; the Person Is a Unit of Body, Mind, and Spirit

Osteopathic medicine views the human body as a complex entity composed of the mind, body, and spirit. It recognizes that the physical state of health can impact the individual's mental and physical functioning [5]. Extensive research has shown a strong connection between the mind and body concerning disease [6-10]. Atopic dermatitis (AD), a chronic inflammatory skin disease, is an excellent example of how the mind-body connection can significantly impact an individual's health. AD is characterized by an immune system imbalance that favors T-helper 2 cells over T-helper 1 cells, which disrupts the skin's natural barrier and leads to a range of symptoms, including pruritus [7]. Furthermore, AD is known to create a positive feedback loop whereby it can lead to exacerbations, often triggered by increased stress levels due to the disease's strong association with the body's stress response and autonomic nervous system dysfunction [11].

Research has highlighted the intricate relationship between neuro-immune interactions and stress in AD patients. Stress triggers the release of acetylcholine, which disrupts cytokine balance and worsens the condition. Peters et al. (2014) conducted a pilot study that demonstrated the profound impact of psychosocial stress on neuroanatomy, leading to neurogenic inflammation and exacerbation of inflammatory skin conditions, including AD [6]. Similarly, a study conducted by Mochizuki et al. (2019) revealed that acute stress may reduce conscious itching sensation while heightening subconscious itching behavior in AD patients, with no such effects observed in healthy controls [7].

Yoshida et al. (2020) conducted a study to investigate the impact of social stress on AD at a genomic level. Specifically, their study focused on the deoxyribonucleic acid methyltransferase 1 gene (DNMT1), vital in deoxyribonucleic acid methylation and gene transcription. Their findings revealed that individuals with low DNMT1 expression had a greater itch score than those with high DNMT1 expression. Furthermore, the study suggested that the downregulation of DNMT1 resulting from social stress could exacerbate the pathology of AD. The study emphasizes the significance of social stress in the development and exacerbation of AD [8].

The connection between the mind and body is bidirectional, and depression can exacerbate the feeling of itchiness in patients with AD [12]. In a study conducted by Talamonti et al. (2021), it was found that AD patients were much more likely to experience depression (56.9%, or 99 out of 174) than the control group of healthy participants (15.7%, or 28 out of 178) (p < 0.001). This highlights how the symptoms associated with AD, particularly itching, can cause psychological distress, leading to depression and a lower quality of life. A significantly higher number of AD patients were suspected to be suffering from depression as well [10].

Chrostowska-Plak et al. (2012) have concluded that pruritus, or itching intensity, is critical in determining the psychosocial well-being of patients with AD. This indicates that patients with AD require effective and continuous antipruritic therapy to improve their quality of life and reduce the potential risk of depression. The study underscores the need to address pruritus in AD management, as it not only impacts patients’ quality of life but also increases the risk of depression [9].

A study by Schut et al. (2013) examined the psychophysiological effects of stress management among patients with AD. They employed cognitive behavioral stress management techniques and measured endocrine stress levels and skin status. The results demonstrated that the group undergoing cognitive behavioral stress management exhibited a notable reduction in endocrine and physiological stress responses. This group maintained a heightened sense of composure and showcased lower salivary cortisol levels even when confronted with acute stressors. These results demonstrate the benefits of addressing both physiological and psychological stress responses in AD therapy. Their conclusions not only validate the hypothesis that stress plays a pivotal role in AD exacerbation but also emphasize the promising role of stress management as a complementary approach to conventional treatments [13].

Dermatologists ought to consider evaluating their patients' stress levels as part of their comprehensive history. It is essential to recognize potential mitigating factors that affect treatment outcomes. They must consider the impact that visible skin diseases can have on patients, physically and psychologically, and how they can affect their everyday lives. Tools such as the Dermatology Life Quality Index can aid in assessing the overall impact of a patient's skin condition on their psychological state. Understanding the powerful influence of emotional and mental factors on overall health and well-being is critical to comprehending the mind-body connection [14].

Tenet 2: The Body Is Capable of Self-Regulation, Self-Healing, and Health Maintenance 

The human body possesses an extraordinary capacity for self-regulation and healing. It can rebuild itself, correct abnormalities, and stimulate growth according to its needs [15]. Expert dermatologists utilize these inherent abilities to promote health and rejuvenation. One notable example is the practice of micro-needling, also known as percutaneous collagen induction (PCI). PCI is a minimally invasive therapeutic treatment that involves the use of fine needles to produce micro punctures in the stratum corneum, which results in controlled skin injury. This controlled injury triggers the body to start a wound healing cascade, which involves various growth factors such as platelet-derived growth factor, transforming growth factors alpha and beta, connective tissue activating protein, connective tissue growth factor, and fibroblast growth factor. This process leads to neovascularization and neocollagenesis, as the growth factors work in unison to form a new intracellular matrix [16].

Acne is a distressing skin condition that can have long-lasting scarring effects following the initial inflammatory response, often posing a daunting challenge for complete eradication. PCI holds promise as an effective solution to improve the appearance of atrophic acne scars. Clinical studies have demonstrated that this treatment method can significantly reduce acne scars and improve scar severity scores. There are several techniques for using PCI to enhance the appearance of scarring. In a study by Leheta et al. (2011), PCI was combined with the focal application of full-concentration trichloroacetic acid (TCA) to atrophic scars resulting from acne. This combined treatment resulted in a remarkable 100% improvement in acne scarring for all patients. Scar severity scores improved by 68.3% (p < 0.001) in patients who underwent four sessions. When combined with TCA, the scar severity scores showed an even more substantial improvement, with a mean of 75.3% (p < 0.001). Although the statistical difference between both groups was insignificant, the outcomes highlight the promising potential of PCI and TCA combination therapy in enhancing the appearance of acne scarring [17].

Leheta et al. (2014) conducted a comparative study to evaluate the efficacy of deep peeling methods in treating atrophic post-acne scars. The study compared phenol versus PCI combined with TCA (20%) in two groups of patients. Both treatment approaches resulted in noticeable improvements in the patient's skin. Statistical analysis of the results revealed that the group treated with PCI combined with 20% TCA showed a striking mean improvement of 69.43% (p < 0.001) in scar severity scores. The treatment was particularly effective for patients with rolling-type scars [18]. 

The study findings demonstrate that deep peeling methods can yield significant improvements in the appearance of atrophic post-acne scars. PCI combined with TCA (20%) was identified as a promising approach, particularly for patients with rolling-type scars. The results of this study provide valuable insights for healthcare professionals and researchers seeking to improve the treatment of atrophic post-acne scars [18].

Leheta et al. (2014) conducted a study aimed at determining the efficacy of combining a 1540 nanometer non-ablative fractional laser with PCI and TCA for the treatment of atrophic scars. The study found that the use of PCI alone resulted in a mean scar severity score of 59.79% (p < 0.001), indicating a significant improvement in the condition. When combined with the non-ablative fractional laser, the mean scar severity score improved to 78.27% (p < 0.001), highlighting the potential of combining PCI with other therapies to further enhance the efficacy of treatment. These findings are significant as they suggest that the use of a non-ablative fractional laser can significantly improve the outcomes of PCI treatment for atrophic scars [19]. The introduction of a laser into the PCI treatment protocol enhances its neocollagenesis effect by stimulating the generation of fresh collagen fibers. This approach has the potential to improve the clinical outcomes of the treatment by promoting the growth of new tissue [20].

Post-burn scars are a significant issue for many patients, and there is a need for effective treatments to improve their quality. Busch et al. (2016) investigated the potential of PCI in combination with non-cultured autologous skin cell suspension (NCASCS) to enhance scar quality and re-pigmentation. The study employed a roller device with 3-millimeter needles to induce microtrauma in the scarred areas, followed by the application of NCASCS. The scar color and appearance were assessed post-operatively using a scar assessment scale, which showed a 50% improvement compared to the baseline (p < 0.05) [21].

The results suggest that the use of PCI with NCASCS can effectively enhance the appearance of post-burn scars. The technique induces microtrauma while preserving the epidermis, which can promote re-pigmentation and improve the overall quality of the scarred tissue. This finding holds significant potential in the clinical management of post-burn scars and warrants further exploration in future research [21].

Post-inflammatory hyperpigmentation, atrophic, and hypertrophic scars are common forms of facial scarring that can significantly impact an individual's self-esteem and quality of life. In recent years, PCI has emerged as an effective treatment for these types of scars. It can enhance the appearance of depressed and elevated facial scars by reducing the height of acne scarring and achieving a clinical response of 100% flattening with the surrounding skin. Compared to traditional surgical and ablative laser procedures, PCI offers a more comfortable treatment experience with minimal discomfort and downtime. As such, PCI is an ideal treatment option for facial scarring, especially for individuals seeking a safe and effective alternative to more invasive surgical or laser procedures [22].

Dermatologists can utilize the body's natural wound-healing cascade to treat various skin conditions such as acne scars, acne, post-traumatic/burn scars, alopecia, skin rejuvenation, hyperhidrosis, and stretch marks [16].

Tenet 3: Structure and Function Are Reciprocally Interrelated 

The skin is the largest organ in the human body and plays a crucial role in the body's immune system and defense mechanisms. It comprises three layers, the epidermis, dermis, and hypodermis, each with unique structures and functions. The epidermis is the outermost layer and includes five sub-layers: the stratum corneum, lucidum, granulosum, spinosum, and basale. The skin's intricate and complex structure is fundamental to function as the body's largest organ. Any disruption in the composition or biochemistry of these layers renders the body vulnerable to pathogens, chemicals, mechanical injury, and ultraviolet damage [23].

Several cutaneous diseases can arise due to defects in the epidermal structure, which ultimately alter the skin's function. One such example is bullous pemphigoid (BP), an autoimmune skin disease that occurs due to the production of antibodies against hemidesmosome autoantigens. Hemidesmosomes are protein complexes that anchor the basal cells of the epidermis to the underlying dermis, and their disruption leads to blisters and erosions on the skin. BP typically affects older individuals and can cause significant morbidity if left untreated [24].

Antibodies that target BP antigens can cause a disruption in the regular connection between the skin layers and the underlying membrane. This disruption can lead to fluid-filled blisters and bullae on the skin's surface. The condition associated with this disruption is known as BP, and it can be a chronic and debilitating autoimmune disease that requires ongoing medical attention and care [25].

The disruption of epidermal attachments can have a significant impact on skin integrity. This can initiate an inflammatory response that attracts reactive oxygen species and proteases, which can cause blister formation. BP implications include bacterial infections such as staphylococcal and streptococcal infections, sepsis, and viral infections like herpes simplex or varicella-zoster virus. The dermal-epidermal junction (DEJ) is a crucial component of skin integrity. This specialized basement membrane connects the epidermis and dermis, which is critical to maintaining skin integrity and function. Any disruption to this layer can compromise the integrity of the skin and lead to blister formation. The loss of structural integrity in the DEJ can weaken the protective layer, making the body more vulnerable to inflammation and injury [26].

In 2018, Russo et al. conducted a study on granzyme B, a protease that immune cells secrete and accumulate in the DEJ in skin disorders characterized by inflammation and blistering. Granzyme B plays a crucial role in causing DEJ separation, particularly in BP, a sub-epidermal skin ailment caused by autoantibodies targeting hemidesmosomes and disrupting the DEJ. In BP cases, the granzyme B levels increase at the DEJ, undermining the junction's structural integrity. The study provides evidence that by using in vitro cleavage assays and amino-terminal oriented mass spectrometry on purified proteins and intact human skin, granzyme B cleaves critical DEJ proteins and separates the epidermis from the dermis, causing physical disruption of the skin architecture. This compromised junction becomes more vulnerable to blistering pathologies. Granzyme B also cleaves the ectodomain of collagen XVII, ultimately producing collagen XVII autoantibodies, resulting in BP. The researchers suggest that Granzyme B could generate autoantibody formation, either directly or indirectly, and could be a therapeutic target for future treatment and prevention of sub-epidermal blistering. There is no established evidence on the relationship between osteopathic manipulation and granzyme B levels in BP. Osteopathic manipulation enhances the performance of the musculoskeletal system. However, more research is required to determine the specific impact of osteopathic manipulation on the immune system, inflammation, and autoimmune conditions like BP, particularly regarding granzyme B levels [27].

Researchers have been studying the various factors contributing to blistering skin diseases for decades. One study conducted by McMillan et al. (1998) shed light on the pathogenesis of junctional epidermolysis bullosa (JEB). This study revealed intrinsic abnormalities within the intracytoplasmic and extracellular basement membrane connections with hemidesmosomes. JEB is a disorder that affects various forms of hemidesmosomes' intracellular functions, which disrupts the connection with keratin intermediate filaments. This subsequently alters the skin's integrity and structure, making it fragile and allowing blisters to form. This pivotal research underscores the complex interplay of molecular components involved in blistering skin disorders [28].

The skin, the body's largest organ, is a prime example of the intricate interplay between structure and function. The skin, comprised of distinct layers, the epidermis, dermis, and hypodermis, houses a range of specific structures and processes. Among these, the DEJ is crucial in maintaining skin integrity and function. Any compromise in the DEJ system can result in the skin's failure to provide adequate support [29].

Tenet 4: Rational Treatment Is Based Upon an Understanding of the Basic Principles of Body Unity, Self-Regulation, and the Interrelationship of Structure and Function 

The etiology of disease is often complex and multifactorial, involving a combination of genetic predisposition, environmental factors, lifestyle choices, and other variables. As such, diagnosing and treating complex medical conditions requires a comprehensive, multidisciplinary approach that considers the many factors contributing to the disease process [29].

A critical aspect of this approach is the careful skin assessment that often the first organ doctors do when evaluating a patient. The skin protects the body against the external environment and can provide important clues about a person's health and well-being. For this reason, all physicians, not just dermatologists, should be trained to perform complete patient assessments that include a thorough examination of the skin [29].

There are several reasons why this is relevant. First, by carefully examining the skin for any changing lesions that may be indicative of skin cancer, physicians can detect these conditions early and provide patients with a better range of treatment options. This can lead to better outcomes and a more favorable prognosis for patients. Additionally, by assessing the skin for cutaneous manifestations of systemic diseases, physicians can identify various health conditions affecting the patient, from autoimmune disorders to infectious diseases. Physicians can provide better care and improve patient outcomes by taking a comprehensive, multidisciplinary approach to patient care that includes careful skin assessment [29].

For pertinent examples, please refer to Table 1.

**Table 1:** Cutaneous manifestations of systemic diseases. The table presented here sheds light on the diverse types of skin abnormalities that can arise due to systemic illnesses. Systemic diseases can cause various cutaneous manifestations on a patient's skin, which can serve as an important diagnostic clue. To ensure a comprehensive evaluation, healthcare professionals must conduct a thorough examination of the skin. Adapted from [29]

Cutaneous manifestation	Description	Systemic disease
Seborrheic keratoses	Acute eruption of many seborrheic keratosis lesions commonly on the trunk (Leser-Trélat sign).	It is a paraneoplastic cutaneous indication of adenocarcinoma of the gastrointestinal tract.
Acanthosis nigricans	Hyperpigmented smooth, velvet-like exophytic eruption on the neck, antecubital, and popliteal fossa.	It is more commonly associated with diabetes and insulin resistance or is, in rare cases, a sign of internal malignancy.
Adenoma sebaceum or ash-leaf spots	Adenoma sebaceum is a firm fibrous/vascular nodule or papule on the face or paranasal area. Ash leaf spots are well-demarcated hypopigmented patches resembling an ash leaf.	Both skin conditions are connected with tuberous sclerosis.
Caput medusae	Prominent varicose veins on the abdomen.	It manifests in portal hypertension.
Butterfly rash	Malar rash involving the facial cheeks and nasal bridge, sparing the nasolabial folds.	It is related to systemic lupus erythematosus.
Gottron’s sign	Multiple erythematous, violaceous, or skin-colored macules or plaques on the dorsum of the hand.	It is associated with dermatomyositis.
Janeway’s lesions	Multiple, non-tender, small hemorrhagic lesions on the palms and soles.	Bacterial endocarditis is linked to it.
Koplik’s spots	Hemorrhagic macules on the oral mucosa may have a yellow pinpoint center near the parotid duct.	These are linked with rubeola, which is commonly known as measles.

The skin is the initial window into a patient's overall well-being. Medical practitioners must be knowledgeable about the possible cutaneous manifestations of systemic processes to provide optimal care. Proper examination of the skin is imperative to enhance diagnostic and therapeutic skills [29].

Discussion

As a multidimensional specialty, dermatology seeks to treat the whole person, not just their skin. Recognizing the potential for better patient outcomes, integrating osteopathic principles and philosophy in dermatology is gaining attention. This paper explored the application of osteopathy in dermatology and the benefits and challenges associated with this holistic approach to skin health [13, 16].

The integumentary system is the largest organ in the human body, and any alterations in its structure can result in cutaneous manifestations of systemic illnesses. It is essential to note that the skin is a barrier against harmful environmental factors and plays a crucial role in regulating body temperature, protecting against fluid loss, and synthesizing vitamin D. Any disruption in its function can significantly impact overall health. For example, BP is an autoimmune skin disorder characterized by blister formation. Researchers have found that hemidesmosomes, which link the epidermis to the dermis, have intrinsic abnormalities that can cause blisters in BP. Granzyme B also plays a role in separating the DEJ and forming autoantibodies that contribute to the development of BP [24-29].

Understanding the interconnectedness of the body's systems, osteopathic dermatologists can assess and address visible and internal health factors to provide comprehensive treatment. One relevant example is the treatment of AD, where patients experience a strong connection between their neuro-immune interactions and stress levels. Acetylcholine's involvement can cause cytokine imbalances to worsen symptoms, while social stress can exacerbate AD and lead to depression. Dermatologists who adopt an osteopathic approach could assess and manage patients' stress levels to enhance overall treatment outcomes [30].

An integration of osteopathic principles and philosophy into dermatology could lead to several potential benefits for patients, including the ones given below.

1. Improved Patient Outcomes 

Recognizing the interrelationship of structure and function allows practitioners to diagnose and treat complex conditions more effectively, leading to better patient outcomes. For example, early detection of skin cancer and other systemic diseases through skin assessments can improve prognosis.

2. A More Holistic Approach 

Integrating osteopathy into dermatology would enable dermatologists to appreciate the interconnectedness of body, mind, and spirit, thus providing more comprehensive patient care.

3. Innovative Treatments ​​​​​​​

Utilizing osteopathic principles and manipulative treatments in techniques like micro-needling, lymphatic drainage, and craniosacral therapy could offer alternative treatment options for patients with skin conditions.

4. Improved Patient-Provider Relationship

Osteopathic dermatologists prioritize listening to patients' concerns and treating them as individuals rather than just a collection of symptoms. This approach can help build stronger relationships between patients and providers, leading to better communication and trust.

5. Preventative Care

By addressing underlying imbalances and promoting overall wellness, osteopathic dermatologists could help prevent the recurrence of certain skin conditions. This approach also reduces the need for medications with potential side effects.

6. Better Management of Stress and Mental Health 

Stress can significantly impact skin health, making it a crucial factor in treating skin conditions. Osteopathic dermatologists can use osteopathic manipulative treatments to target specific body areas affected by stress, such as the neck, shoulders, and scalp.

7. Individualized Care Plans

Dermatologists who practice osteopathy take a comprehensive approach to treating skin conditions, considering all aspects of a patient's health and lifestyle. This holistic approach enables them to create personalized treatment plans that address the underlying causes of skin problems.

8. Continued Education and Research

Osteopathic dermatologists are committed to staying current with the latest advancements in osteopathic medicine and dermatology. They also research further to understand the relationship between osteopathy and skin health, leading to more evidence-based treatments.

9. Accessible and Affordable Care

Osteopathic dermatologists strive to make their services accessible and affordable for patients of all backgrounds. Some may offer sliding scale fees or accept insurance to ensure patients receive the care they need without financial barriers. Additionally, osteopathic manipulative treatments are non-invasive and do not require expensive equipment, making them a cost-effective option for patients.

10. Patient Education and Empowerment

Along with providing treatment, osteopathic dermatologists educate their patients on maintaining healthy skin through lifestyle changes and self-care practices. This empowers patients to take an active role in their health and well-being.

11. Preventative Care ​​​​​​​

Osteopathic dermatologists strongly emphasize preventive care, seeking to recognize and tackle potential issues before they escalate into more severe or long-lasting problems. By addressing underlying imbalances and promoting overall wellness, osteopathic treatments can help prevent future skin problems.

12. Continued Growth and Recognition 

As osteopathic dermatology grows, it becomes increasingly recognized as a valuable and practical approach to skin health. Osteopathic dermatologists are gaining recognition for their expertise and unique perspective in the medical community.

Considerations for future research

This review paper emphasizes the importance of skin examination and its significance in all patient encounters. Although dermatologists routinely assess skin, it may not be considered the most optimal use of time for medical professionals from other specialties, primarily if their patient's primary concern is unrelated to dermatology. Educating practitioners on the significance of complete skin examinations for all patients and how to perform these examinations as thoroughly as a dermatologist would help improve the situation. Such education should include detailed instructions on skin cancer detection and pattern recognition when finding connections between cutaneous manifestations and systemic disease.

An osteopath-trained dermatologist can use somatic findings to link structure and function and then use osteopathic manipulative treatment to enhance homeostatic mechanisms. A successful treatment could eliminate the need for systemic therapy and a more expensive and time-consuming workup. Another area of future consideration is exploring the benefits of osteopathic manipulation for dermatologic conditions.

## Conclusions

The foundations of dermatology and osteopathic medicine share a common consideration: the importance of physical touch. Palpation, or examining a patient through touch, is emphasized in both disciplines. According to osteopathic principles, hands are essential diagnostic tools for assessing pressure, blanching, edema, skin fragility or shearing, and other factors when evaluating cutaneous diseases. Palpation and physical touch are not only used for diagnostic purposes but also for therapeutic ones. Osteopathic manipulative treatment can serve as an adjunct therapy to improve the treatment of many dermatologic diseases. By comprehending cutaneous disease and functioning and how it is connected to systemic disease states, physicians can use an osteopathic approach to treat the disease from the inside out, reinforcing the four core osteopathic tenets. Treating the whole person requires meticulous attention to detail and understanding the patient on all levels, including physical, psychological, and emotional. An osteopathic approach leads to more accurate diagnoses and successful treatments of dermatologic conditions since the foundation of dermatology is based on osteopathic principles.
